# Association between urinary polycyclic aromatic hydrocarbon metabolites and diabetes mellitus among the US population: a cross-sectional study

**DOI:** 10.1093/inthealth/ihac029

**Published:** 2022-06-25

**Authors:** Manthar Ali Mallah, Til Bahadur Basnet, Mukhtiar Ali, Fuwei Xie, Xiang Li, Feifei Feng, Wei Wang, Pingping Shang, Qiao Zhang

**Affiliations:** Department of Toxicology and Occupational health, College of Public Health, Zhengzhou University, Zhengzhou 450001, China; Department of Epidemiology and Health Statistics, School of Public Health, Fujian Medical University, Fujian 350122, China; Department of Chemical Engineering, Quaid-e-Awam University of Engineering, Science & Technology, Nawabshah 67480, Sindh, Pakistan; Key Laboratory of Tobacco Chemistry, Zhengzhou Tobacco Research Institute, CNTC, Zhengzhou, China; Key Laboratory of Tobacco Chemistry, Zhengzhou Tobacco Research Institute, CNTC, Zhengzhou, China; Department of Toxicology and Occupational health, College of Public Health, Zhengzhou University, Zhengzhou 450001, China; Department of Toxicology and Occupational health, College of Public Health, Zhengzhou University, Zhengzhou 450001, China; Key Laboratory of Tobacco Chemistry, Zhengzhou Tobacco Research Institute, CNTC, Zhengzhou, China; Department of Toxicology and Occupational health, College of Public Health, Zhengzhou University, Zhengzhou 450001, China

**Keywords:** diabetes mellitus, environmental contaminate, polycyclic aromatic hydrocarbon, urinary PAH

## Abstract

**Background:**

The primary aim of this study is to examine the association between urinary polycyclic aromatic hydrocarbons (PAHs) and diabetes mellitus (DM) among the US population.

**Methods:**

We used data from the National Health and Nutritional Examination Survey 2003–16, which is a nationally representative population-based survey of the US non-institutionalized population. Logistic regression analysis was performed to evaluate the association between urinary PAHs and the prevalence of DM using odds ratios (ORs) and 95% confidence intervals (CIs).

**Results:**

The study sample including 13 792 individuals ≥18 y of age. The average ages of the three PAH tertiles were 42.56±19.67, 42.21±19.51 and 43.39±17.99 y. An increased risk of DM was found with increased odds for the second (OR 1.56 [95% CI 1.36 to 1.79]) and third tertile (OR 1.79 [95% CI 1.55 to 2.06)] of urinary PAH as compared with the first tertile. Similarly, higher chances of DM were observed in the second (men: OR 1.42 [95% CI 1.18 to 1.71]; women: OR 1.76 [95% CI 1.44 to 2.14]) and third tertile (men: OR 1.69 [95% CI 1.38 to 2.08]; women: OR 1.79 [95% CI 1.46 to 2.19]) of urinary PAHs as compared with the first tertile in both men and women.

**Conclusions:**

A population-based cross-sectional study found a positive association between urinary PAHs and DM in the US population.

## Introduction

Diabetes is a chronic disease that is one of the most common causes of disease burden and death worldwide.^1,2^ It is becoming more common, with the International Diabetes Federation (IDF) projecting that the number of diabetic patients will increase dramatically to 591.9 million by the year 2035.^3^ It may impose significant costs on the healthcare system. In 2015, the overall cost of diabetes-related healthcare expenditures was US${\$}$1.31 trillion, or 1.8 percent of worldwide gross domestic product.^4^ Similarly, by 2030 this expenditure is expected to increase to US${\$}$2.2 trillion.^5^ In addition to well-documented risk factors, including age, unhealthy dietary patterns, physical inactivity, smoking and obesity,^6^ recent findings have suggested that work-related and environmental factors like noise, air pollution, shift work and electromagnetic fields^7^ may have an influence on the progression of diabetes. In contrast, environmental contaminants are well known to be linked to a variety of chronic diseases.^8^

Polycyclic aromatic hydrocarbons (PAHs) are lipid-soluble contaminants produced by cigarette smoking, incomplete biomass, fossil fuels combustion, preparation of grilled and smoked foods, industrial procedures and volcanoes and forest fires.^9^ PAHs are widespread pollutants that may be found in air, water, soil and sediments^10^ due to their physicochemical characteristics, such as high melting and boiling points and low vapor pressure.^11^ Although inhalation is the most common route of PAH exposure, PAHs can be inhaled, absorbed via the skin or consumed in work-related and environmental situations.^12^ Nonetheless, based on the features stated above, PAHs are among the top 10 compounds on the priority list of hazardous materials.^12^ PAHs are converted in the body to monohydroxylated metabolites of PAH (OH-PAHs), which are mostly excreted in the urine in the first few hours after exposure.^13^ Urinary OH-PAHs measurement is a useful biomarker for determining recent PAH exposure via multiple pathways.^14^

Exposure to PAHs is likely influenced by non-modifiable risk factors, such as age, sex and race, as well as occupational risks, active smoking and/or passive smoking exposure. Being overweight or obese is a risk factor for diabetes^15^ and highly lipid-soluble PAHs.^16^ However, individuals with a low body mass index (BMI) are unlikely to be exposed to PAHs differently than those with a high BMI. The liver and kidney predominantly process PAHs after being eaten, breathed or absorbed via the skin and subsequently eliminated in bile and urine.^15^ Furthermore, PAHs have been detectable in virtually all internal organs, particularly those with large quantities of adipose tissue.^9^ Individual risk factors and comorbidities can operate synergistically with the lipophilic characteristics of PAHs, fluctuating with exposure length, exposure route and concentration to increase the severity of effects on the human body.^9,15^ Because PAHs are retained in adipose tissue until they are evacuated by regular bladder and gastrointestinal activities,^15^ PAHs may be more persistent in people with a higher BMI, which might impact their chances of developing diabetes compared with people with a lower BMI.

Previous research has linked ambient air pollution to diabetes incidence, diabetes-related hospitalization and diabetes-related deaths, including a link between permanent organic contaminants and diabetes prevalence.^17,18^ In addition, many studies have examined the impacts of PAHs on people. These studies found that PAH exposure increases the risk of diseases such as cancer, DNA damage, cardiovascular diseases and metabolic syndrome through a variety of pathways.^19,20^ Several studies have looked at the possible relationship between urinary PAH metabolites and diabetes.^15,21–25^ Despite this, an association remains uncertain due to conflicting findings across research. Therefore the primary aim of this study is to examine the association between urinary PAHs and diabetes mellitus (DM) in the US population.

## Methods

### Study population

**Figure 1.**
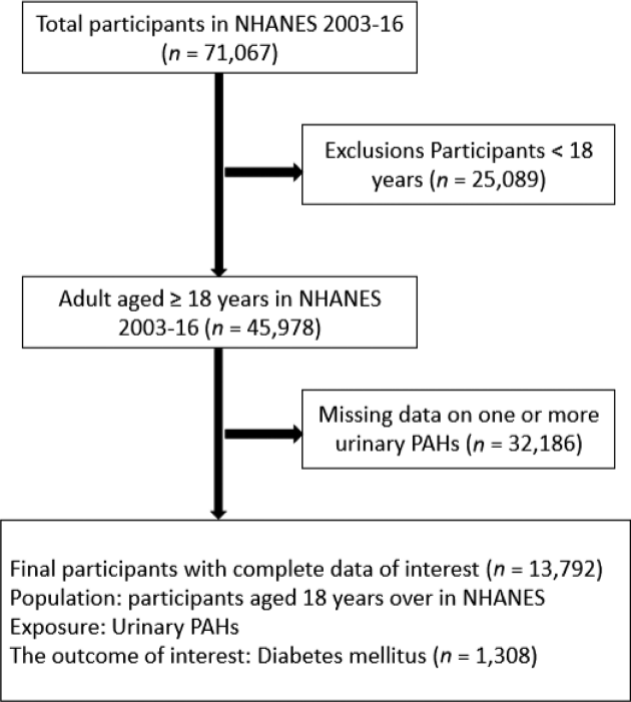
Eligible participants and those included in the analyses of the association between urinary PAHs and DM among the US population.

The National Health and Nutritional Examination Survey (NHANES), a population-based survey, is a nationally representative study that was utilized to compile the data from 2003 to 2016 for the present study. It consists of a series of surveys created by the National Center for Health Statistics (NCHS) of the Centers for Disease Control and Prevention (CDC) to continually monitor the health status of the non-institutionalized civilian population in the USA.^26^ The NHANES program has included a series of surveys focusing on different demographic groups or health issues since its beginning in the early 1960s. A cross-sectional study was planned to determine the degree of the association between urinary PAHs and DM. We used data from the 2003–2004, 2005–2006, 2007–2008, 2009–2010, 2011–2012, 2013–2014 and 2015–2016 (seven) data cycles for our study. In all, 71 067 participants were included in this study throughout seven cycles, with 45 978 of them being ≥18 y of age. The urinary PAH metabolites were only examined in an NHANES subsample (n=13 792). Participants who missed information on PAHs and were <18 y of age were omitted from the final design. Consequently, 13 792 participants of the NHANES 2003–2016 were included in the final analyses (Figure 1).

On the day of the physical examination, all participants completed the questionnaires and underwent a basic physical examination as well as provided blood and urine samples. Trained professionals collected data on demographic parameters, employment history, personal and family medical history and lifestyle behaviours, such as smoking and alcohol consumption, using structured questionnaires. The NHANES procedure was approved by the NCHS Institutional Review Committee and signed informed consent forms were acquired.

### Data collection

A structured medical condition questionnaire was administered for a wide array of health conditions, including DM, during the personal interview. ‘Has a doctor or other health professional ever informed you that you have diabetes mellitus?’ If a participant replied ‘yes’, she/he was classified as a DM case.^27^ Each participant's morning urine sample was collected in a sterile tube and were preserved at −20°C until they were utilized. Six urinary PAH metabolites were frequently available and tested in NHANES 2003–2016, including 1-hydroxynaphthalene, 2-hydroxynaphthalene, 2-hydroxyfluorene, 3-hydroxyfluorene, 1-hydroxyphenanthrene and 1-hydroxypyrene, using enzymatic hydrolysis of urine followed by removal, derivation and investigation using capillary gas chromatography and high-resolution mass spectrometry (GC-HRMS). The same unit, ng/L, was used to measure all of the metabolites.^28^ The lowest point in the calibration curve that has been identified to generate a signal:noise ratio (S:N) ≥3 was defined as the limit of detection (LOD) for urinary PAH metabolites.^20^ A structured questionnaire administered during a home interview collected sociodemographic information such as age (years), gender (men/women), marital status, ethnicity, education, work type, housing type, number of people in the household, vigorous work activity, moderate work activity, vigorous recreational activities, moderate recreational activities, smoking and drinking.^29^ Data on anthropometric, physical and laboratory parameters were gathered during the medical centre assessment. Height and weight were recorded without shoes and in light indoor clothing. BMI was calculated as kilograms per square meter (kg/m^2^). Using BMI cut-off points, participants were classified into three groups: <25, 25–29.9 and ≥30 kg/m^2^. The fasting serum lipid profile, comprising total cholesterol (TC), triglycerides (TG), low-density lipoprotein (LDL) and high-density lipoprotein (HDL) was determined using a biochemical blood analyser.

### Statistical analyses

For normally distributed continuous variables, an analysis of variance was performed to investigate variations in participant characteristics by tertiles of total PAHs. The χ^2^ test was used to compare the frequencies of the categorical variables. Urinary PAH metabolites concentration (ng/L) was adjusted by the corresponding urinary creatinine concentration (mg/dL), divided, and then multiplied by 0.01. Total PAHs was divided into tertile 1, tertile 2 and tertile 3. Logistic regression analysis with confounder adjusted odds ratios (ORs) and 95% confidence intervals (CIs) was performed to evaluate the association between urinary PAHs and the prevalence of diabetes. Model 1 was unadjusted, model 2 was adjusted for age (years) and gender (men/women) and model 3 was adjusted for model 2 plus marital status, ethnicity, education, work type, housing type, number of people in the household, vigorous work activity, moderate work activity, vigorous recreational activities, moderate recreational activities, smoking, drinking and BMI. All analyses were performed using SPSS version 25.0 (IBM, Armonk, NY, USA). Statistical significance was defined as a two-tailed p-value <0.05.

## Results

### Characteristics of participants

The study sample consisted of 13 792 individuals ≥18 y of age; 6866 were men and 6926 were women. The mean age of all participants was 42.72±19.08. In our study, we analysed the participants’ characteristics for three different tertiles; the average ages of the three PAH tertiles were 42.56±19.67, 42.21±19.51 and 43.39±17.99 y, as shown in Table 1. The age, gender, marital status, ethnicity, education, work type, housing type, number of people in the household, vigorous recreational activities, moderate recreational activities, smoking, drinking, BMI, HDL, TC, TG and diabetes were significantly different (p<0.05) between the PAH tertiles (Table 1). No significant differences in vigorous work activity, moderate work activity and LDL were observed across the tertiles of urinary PAH (p>0.05).

**Table 1.** Demographic and health characteristics of the study participants by urinary PAH quartiles among the US adult population

		Ʃ PAHs (ng/g creatinine)*0.01				
Characteristics	All participants (N=13 792)	≤0.52 (n=4604)	0.53–1.29 (n=4594)	≥1.30 (n=4594)	Statistic values	P-Value
Age (years), mean ±SD	42.72±19.08	42.56±19.67	42.21±19.51	43.39±17.99	4.647^a^	0.01
Age group (years), n (%)						
≥18–39	7090 (51.4)	2451 (53.2)	2437 (53.0)	2202 (47.9)	76.299^b^	<0.001
40–59	3371 (24.4)	1003 (21.8)	1040 (22.6)	1328 (28.9)		
60–≥80	3331 (24.2)	1150 (25.0)	1117 (24.3)	1064 (23.2)		
Gender, n (%)						
Male	6866 (49.8)	2622 (57.0)	2084 (45.4)	2160 (47.0)	144.553^b^	<0.001
Female	6926 (50.2)	1982 (43.0)	2510 (54.6)	2434 (53.0)		
Marital status, n (%)						
Married	6323 (50.4)	2281 (54.7)	2125 (52.3)	1917 (44.4)	113.721^b^	<0.001
Divorced	2644 (21.1)	738 (17.7)	830 (20.4)	1076 (24.9)		
Single	3590 (28.6)	1151 (27.6)	1110 (27.3)	1329 (30.7)		
Ethnicity, n (%)						
Mexican American	2356 (17.1)	688 (149)	964 (21.0)	704 (15.3)	121.562^b^	<0.001
Other Hispanic	1236 (9.0)	361 (7.8)	445 (9.7)	430 (9.4)		
Non-Hispanic white	5750 (41.7)	2041 (44.3)	1704 (37.1)	2005 (43.6)		
Non-Hispanic Black	3051 (22.1)	1019 (22.1)	981 (21.4)	1051 (22.9)		
Other race including multiracial	1399 (10.1)	495 (10.8)	500 (10.9)	404 (8.8)		
Education level, n (%)						
<9th grade	1418 (11.6)	379 (9.5)	527 (13.3)	512 (12.0)	437.26^b^	<0.001
9th–11th grade	1758 (14.4)	430 (10.7)	514 (13.0)	814 (19.1)		
High school graduate	2867 (23.4)	828 (20.7)	900 (22.7)	1139 (26.7)		
College degree	3508 (28.7)	1175 (29.4)	1099 (27.7)	1234 (29.0)		
College and above	2670 (21.8)	1187 (29.7)	925 (23.3)	558 (13.1)		
Work type, n (%)						
Employee of a private company	5447 (74.9)	1790 (72.0)	1843 (74.6)	1814 (74.6)	40.688^b^	<0.001
Federal government employee	180 (2.5)	78 (3.1)	66 (2.7)	36 (1.6)		
State government employee	444 (6.1)	184 (7.4)	148 (6.0)	112 (4.8)		
Local government employee	445 (6.1)	170 (6.8)	153 (6.2)	122 (5.3)		
Self-employed	695 (9.6)	243 (9.8)	243 (9.8)	209 (9.0)		
Working without pay in farming	24 (0.3)	8 (0.3)	6 (0.2)	10 (0.4)		
Housing type, n (%)						
Own	3710 (56.4)	1170 (62.0)	1322 (57.0)	1218 (51.4)	51.829^b^	<0.001
Rent	2859 (43.5)	717 (38.0)	993 (42.8)	1149 (48.5)		
People in household, n (%)						
1–2	4757 (39.3)	1602 (41.4)	1501 (36.5)	1654 (40.3)	30.306^b^	<0.001
3–4	4276 (35.4)	1376 (35.5)	1494 (36.4)	1406 (34.2)		
5–6	1455 (12.0)	442 (11.4)	531 (12.9)	482 (11.7)		
≥7	1601 (13.2)	454 (11.7)	582 (14.2)	35.3 (13.8)		
Vigorous work activity, n (%)						
Yes	1134 (19.0)	326 (18.7)	401 (18.9)	407 (19.5)	3.446^b^	0.751
No	4823 (80.9)	1420 (81.3)	1725 (81.1)	1678 (80.4)		
Moderate work activity, n (%)						
Yes	2111 (35.4)	594 (34.0)	793 (37.3)	724 (34.7)	9.226^b^	0.161
No	3844 (64.5)	1151 (65.9)	1333 (62.7)	1360 (65.2)		
Vigorous recreational activities, n (%)						
Yes	1438 (24.1)	466 (26.7)	513 (24.1)	459 (22.0)	13.131^b^	0.011
No	4521 (75.9)	1281 (73.3)	1614 (75.9)	1626 (77.9)		
Moderate recreational activities, n (%)						
Yes	2391 (40.1)	745 (42.6)	857 (40.3)	789 (37.8)	10.44^b^	0.034
No	3567 (59.8)	1001 (57.3)	1269 (59.7)	1297 (62.2)		
Smoking status, n (%)						
Yes	2253 (38.3)	121 (9.1)	375 (24.8)	1757 (58.1)	1100.979^b^	<0.001
No	3622 (61.7)	1214 (90.9)	1140 (75.2)	1268 (41.9)		
Drinking status, n (%)						
Yes	8204 (71.7)	2627 (70.1)	2589 (69.4)	2988 (75.3)	46.541^b^	<0.001
No	3234 (28.3)	1118 (29.8)	1141 (30.6)	975 (24.6)		
BMI (kg/m^2^), n (%)						
<25	4734 (34.8)	1604 (35.3)	1529 (33.7)	1601 (35.3)	9.604^b^	0.048
25–29.9	4220 (31.0)	1454 (32.0)	1391 (30.7)	1375 (30.3)		
≥30	4666 (34.3)	1492 (32.8)	1615 (35.6)	1559 (34.4)		
Cholesterol level, mean±SD						
HDL (mg/dL)	54.03±15.42	53.6915.14	56.0216.02	52.57±15.04	6.159^a^	0.002
LDL (mg/dL)	111.90±5.37	112.16±34.71	110.44±35.08	113.11±36.34	2.812^a^	0.06
TC (mg/dL)	190.86±42.27	190.43±42.16	189.71±41.46	192.44±43.14	4.856^a^	0.008
TG (mg/dL)	129.0±125.63	125.83±103.83	123.34±133.48	138.28±137.99	7.959^a^	<0.001
Diabetes, n (%)						
Yes	1308 (10.8)	395 (10.2)	444 (10.8)	469 (11.4)	11.933^b^	0.003
No	10781 (89.2)	3479 (89.8)	3664 (89.2)	3638 (88.6		

Ʃ PAH: total sum of all PAH metabolites; SD: standard deviation.

^a^χ^2^ value.

b F value.

### Association of urinary PAHs with DM

Table 2 shows the associations between urinary PAHs and the increased prevalence of diabetes. The second and third tertiles were significantly associated with an increased prevalence of diabetes. A positive association was found for the second and third tertiles of urinary PAHs and the prevalence of diabetes (OR 1.56 [95% CI 1.36 to 1.79] and OR 1.79 [95% CI 1.55 to 2.06], respectively, with p<0.05. Similarly, men and women had a significantly positive association between the second and third tertiles of urinary PAHs with DM (men: OR 1.42 [95% CI 1.18 to 1.71], OR 1.76 [95% CI 1.44 to 2.14] and women: OR 1.69 [95% CI 1.38 to 2.08], OR 1.79 [95% CI 1.46 to 2.19]; model 1; Table 2). Moreover, after adjustment for age (years) and gender (men/women), an increased prevalence risk for diabetes was observed in both (second and third) tertiles. Likewise, in both men and women, a positive association was observed in the third tertiles of urinary PAHs with a prevalence of diabetes; however, no significant relationship was found in the second tertiles of both sexes (model 2; Table 2). Furthermore, after adjustment for age (years), gender (men, women), marital status, ethnicity, education, work type, housing type, number of people in the household, vigorous work activity, moderate work activity, vigorous recreational activities, moderate recreational activities, smoking, drinking and BMI, there was no association observed between urinary PAH tertiles and the prevalence of diabetes. Additionally, a significant relationship was found among men and women (model 3; Table 2).

**Table 2.** Association between urinary PAH levels and diabetes among the US adult population

	Diabetes (n=1308)					
PAH quartiles	Model 1		Model 2		Model 3	
All participants	OR (95% CI)	P-Value	OR (95% CI)	P-Value	OR (95% CI)	P-Value
Tertile 1 (≥0.52) (n=4604)	Reference		Reference		Reference	
Tertile 2 (0.53–1.29) (n=4594)	1.56 (1.36 to 1.79)	<0.001	1.56 (1.36 to 1.79)	<0.001	1.44 (0.83 to 2.52)	0.195
Tertile 3 (≥1.30) (n=4594)	1.79 (1.55 to 2.06)	<0.001	1.79 (1.55 to 2.06)	<0.001	1.28 (0.76 to 2.19)	0.356
Men						
Tertile 1 (≥0.52) (n=2622)	Reference		Reference		Reference	
Tertile 2 (0.53–1.29) (n=2084)	1.42 (1.18 to 1.71)	<0.001	1.15 (0.94 to 1.39)	0.168	1.59 (0.85 to 2.97)	0.149
Tertile 3 (≥1.30) (n=2160)	1.76 (1.44 to 2.14)	<0.001	1.36 (1.12 to 1.66)	0.003	1.79 (0.96 to 3.37)	0.068
Women						
Tertile 1 (≥0.52) (n=1982)	Reference		Reference		Reference	
Tertile 2 (0.53–1.29) (n=2510)	1.69 (1.38 to 2.08)	<0.001	1.20 (0.97 to 1.49)	0.091	1.09 (0.30 to 3.98)	0.889
Tertile 3 (≥1.30) (n=2434)	1.79 (1.46 to 2.19)	<0.001	1.10 (0.88 to 1.37)	0.387	0.61 (1.86 to 2.01)	0.419

PAH (ng/g creatinine)*0.01. Logistic regression was used.

Model 1 unadjusted. Model 2 adjusted for age and gender. Model 3 adjusted for age, gender, marital status, ethnicity, education level, work type, housing type, number of people in the household, vigorous work activity, moderate work activity, vigorous recreational activities, moderate recreational activities, smoking, drinking and BMI.

### Association of urinary PAH metabolites with DM

Table 3 shows the association between six urinary PAH metabolites and the prevalence of diabetes. We used the logistic regression method to analyse the association between PAH metabolites and diabetes. The results indicated that the increased diabetes prevalence was observed across the second and third tertiles of 3-hydroxyfluorene (OR 1.28 [95% CI 1.12 to 1.47] and OR 1.32 [95% CI 1.14 to 1.51]) and the second and third tertiles of 1-hydroxypyrene (OR 1.56 [95% CI 1.36 to 1.79] and OR 1.79 [95% CI 1.55 to 2.06]), with p<0.05 (model 1; Table 3). Similarly, after adjustment for age (years) and gender (men/women), a positive association was observed between the second and third tertiles of 3-hydroxfluorene, the second and third tertiles of 1-hydroxypyrene and both tertiles of 1-hydroxyphenanthrene with diabetes, with p<0.05 (model 2; Table 3). Additionally, after adjustment for marital status, ethnicity, education, work type, housing type, number of people in the household, vigorous work activity, moderate work activity, vigorous recreational activities, moderate recreational activities, smoking, drinking and BMI in model 2 (and model 3), we did not find a statistically significant association between the levels of each PAH metabolite and the prevalence of diabetes, with p>0.05.

**Table 3.** Association between PAH metabolite levels and diabetes among the US adult population

	Diabetes (n=1308)					
PAH biomarkers	Model-1		Model 2		Model 3	
	OR (95% CI)	P-Value	OR (95% CI)	P-Value	OR (95% CI)	P-Value
1-Hydroxynaphthalene						
Tertile 1 (≤0.1) (n=4129)	Reference		Reference		Reference	
Tertile 2 (0.11–0.39) (n=3977)	0.85 (0.73 to 0.98)	0.024	1.02 (0.89 to 1.18)	0.808	0.72 (0.41 to 1.26)	0.244
Tertile 3 (≥0.4) (n=3979)	0.78 (0.68 to 0.90)	0.001	0.99 (0.86 to 1.16)	0.986	1.04 (0.61 to 1.78)	0.886
2-Hydroxynaphthalene						
Tertile 1 (≤0.28) (n=3738)	Reference		Reference		Reference	
Tertile 2 (0.29–0.73) (n=4144)	0.93 (0.81 to 1.08)	0.353	0.78 (0.68 to 0.91)	0.001	1.14 (0.60 to 2.18)	0.681
Tertile 3 (≥0.74) (n=4203)	0.94 (0.81 to 1.08)	0.378	0.75 (0.64 to 0.87)	<0.001	1.09 (0.59 to 2.01)	0.783
3-Hydroxyfluorene						
Tertile 1 (≤0.01) (n=4022)	Reference		Reference		Reference	
Tertile 2 (0.01–0.02) (n=4079)	1.28 (1.12 to 1.47)	<0.001	1.23 (1.06 to 1.42)	0.005	1.17 (0.63 to 2.14)	0.623
Tertile 3 (≥0.2) (n=3984)	1.32 (1.14 to 1.51)	<0.001	1.19 (1.03 to 1.37)	0.02	1.02 (0.59 to 1.76)	0.939
2-Hydroxyfluorene						
Tertile 1 (≤0.02) (n=4071)	Reference		Reference		Reference	
Tertile 2 (0.03–0.03) (n=4037)	1.11 (0.96 to 1.27)	0.152	1.13 (0.98 to 1.31)	0.088	1.03 (0.58 to 1.84)	0.91
Tertile 3 (≥0.4) (n=3977)	1.09 (0.95 to 1.26)	0.192	1.08 (0.94 to 1.25)	0.261	1.19 (0.69 to 2.05)	0.535
1-Hydroxypyrene						
Tertile 1 (≤0.01) (n=3715)	Reference		Reference		Reference	
Tertile 2 (0.02–0.02) (n=4159)	1.56 (1.36 to 1.79)	<0.001	1.18 (1.02 to 1.36)	0.025	1.45 (0.83 to 2354)	0.191
Tertile 3 (≥0.03) (n=4211)	1.79 (1.55 to 2.06)	<0.001	1.22 (1.05 to 1.42)	0.009	1.30 (0.76 to 2.23)	0.331
1-Hydroxyphenanthrene						
Tertile 1 (≤0.01) (n=4135)	Reference		Reference		Reference	
Tertile 2 (0.01–0.02) (n=4028)	1.08 (0.94 to 1.24)	0.266	1.18 (1.02 to 1.36)	0.029	1.36 (0.80 to 2.31)	0.255
Tertile 3 (≥0.03) (n=3922)	1.07 (0.93 to 1.23)	0.324	1.24 (1.07 to 1.44)	0.004	0.84 (0.50 to 1.41)	0.516

PAH (ng/g creatinine)*0.01. Logistic regression was used.

Model 1 unadjusted. Model 2 adjusted for age and gender. Model 3 adjusted for age, gender, marital status, ethnicity, education level, work type, housing type, number of people in the household, vigorous work activity, moderate work activity, vigorous recreational activities, moderate recreational activities, smoking, drinking and BMI.

### Stratification analysis of urinary PAHs and the prevalence of DM

We performed subgroup analysis stratified by age (groups), gender (men/women), marital status (married, divorced, single), housing type (owned/rented), vigorous work activity (yes/no), moderate work activity (yes/no), vigorous recreational activities (yes/no), moderate recreational activities (yes/no), smoking status (yes/no), drinking status (yes/no) and BMI. In the age group ≥18–39 y, the second and third tertiles of urinary PAHs were significantly associated with the prevalence of diabetes (OR 1.54 [95% CI 1.15 to 2.07] and 1.32 [95% CI 0.99 to 1.76]), respectively, with p<0.05. A positive association was also observed between the second and third tertiles of urinary PAHs and diabetes in both men and women (men: OR 1.42 [95% CI 1.18 to 1.71] and 1.76 [95% CI 1.44 to 2.14]; women: 1.69 (95% CI 1.38 to 2.08) and 1.79 (95% CI 1.46 to 2.19), with p<0.05, respectively]; Table 4).

**Table 4.** Association between PAH and diabetes stratified by variables among the US population

	PAH tertile 2(n=4594)		PAH tertile 3(n=4594)	
Variables	OR (95% CI)	P-Value	OR (95% CI)	P-Value
Age group (years)				
≤18–39	1.54 (1.15 to 2.07)	0.004	1.32 (0.99 to 1.76)	0.051
40–59	1.11 (0.84 to 1.48)	0.449	1.11 (0.85 to 1.45)	0.451
60–≥80	1.14 (0.93 to 1.39)	0.205	1.48 (1.17 to 1.87)	0.001
Gender				
Male (n=6864)	1.42 (1.18 to 1.71)	<0.001	1.76 (1.44 to 2.14)	<0.001
Female (n=6924)	1.69 (1.38 to 2.08)	<0.001	1.79 (1.46 to 2.19)	<0.001
Marital status				
Married	1.36 (1.13 to 1.63)	0.001	1.51 (1.24 to 1.84)	0.001
Divorced	1.49 (1.15 to 1.94)	0.003	1.96 (1.50 to 2.55)	<0.001
Single	1.64 (1.11 to 2.43)	0.013	1.25 (0.88 to 1.77)	0.212
Housing type				
Own	1.55 (1.22 to 1.97)	<0.001	1.59 (1.24 to 2.04)	<0.001
Rent	1.39 (1.02 to 2.74)	0.036	2.00 (1.46 to 2.74)	<0.001
Vigorous work activity				
Yes	1.29 (0.78 to 2.12)	0.312	1.15 (0.72 to 1.84)	0.567
No	1.59 (1.28 to 1.98)	<0.001	2.06 (1.64 to 2.59)	<0.001
Moderate work activity				
Yes	1.32 (0.93 to 1.88)	0.123	1.40 (0.98 to 1.99)	0.061
No	1.67 (1.31 to 2.13)	<0.001	2.11 (1.64 to 2.72)	<0.001
Vigorous recreational activities				
Yes	1.83 (1.15 to 2.89)	0.01	1.49 (0.96 to 2.33)	0.077
No	1.48 (1.18 to 1.84)	0.001	1.94 (1.54 to 2.45)	<0.001
Moderate recreational activities				
Yes	1.48 (1.08 to 2.03)	0.014	2.01 (1.43 to 2.81)	<0.001
No	1.58 (1.22 to 2.05)	<0.001	1.77 (1.37 to 2.29)	<0.001
Smoking status				
Yes	1.05 (0.58 to 1.90)	0.857	1.16 (0.68 to 1.98)	0.593
No	1.48 (1.18 to 1.86)	0.001	2.34 (1.83 to 2.96)	<0.001
Drinking status				
Yes	1.41 (1.18 to 1.69)	0.001	1.62 (1.35 to 1.94)	0.001
No	1.74 (1.37 to 2.22)	<0.001	1.86 (1.43 to 2.43)	<0.001
BMI (kg/m^2^)				
<25	1.97 (1.37 to 2.86)	<0.001	1.78 (1.27 to 2.51)	0.001
25–29.9	1.11 (0.85 to 1.44)	0.444	1.33 (1.01 to 1.75)	0.043
≥30	1.61 (1.34 to 1.95)	<0.001	1.59 (1.31 to 1.94)	<0.001

PAH (ng/g creatinine)*0.01.

PAH tertile 1 is the reference group.

Logistic regression was used for stratification.

## Discussion

Our findings demonstrate that increased levels of urinary PAHs were positively associated with DM in the US general population. In both sexes, the second and third tertiles of PAH levels compared with the first tertile were strongly linked with an increased OR for DM. Moreover, after adjustment for age and gender, the higher tertiles of urinary PAH levels were associated with DM, but interestingly, statistical significance was found in the higher tertile of urinary PAHs in males only. To our knowledge this is the first large-scale nationwide population-based epidemiological study showing the association between urinary PAHs and DM. Our study also found a positive association of 3-hydroxyfluorene and 1-hydroxypyrene with DM, after adjustment for age and gender. Thus our findings support prior research linking diabetes with PAH exposure^21^ and other persistent organic pollutants.^30^

The present study examined the association of urinary PAH values with the prevalence of diabetes and found significance in both men and women, even after adjustment for age. Nevertheless, after adjustment for confounding factors, the relationship was not confirmed. The majority of the confounding factors adjusted for in this study are DM risk factors, thus further adjustment for these covariates is required and should be undertaken carefully. Moreover, the findings of this study suggest that smoking and drinking habits, work type, exercise types and BMI may all influence the link between PAHs and the prevalence of DM. Furthermore, the findings of this research should be confirmed in a cohort or longitudinal studies.

According to our findings, a higher OR for diabetes was observed among the individuals exposed to 1-hydroxynaphthalene, 2-hydroxynaphthalene and 2-hydroxyfluorene metabolites, but we did not find significant results. However, an insignificant relationship was observed for 1-hydroxynaphthalene, 2-hydroxynaphthalene and 2-hydroxyfluorene metabolites. Although 1-hydroxynaphthalene is commonly used as an indicator of major metabolites of PAH exposure,^31^ the true diagnostic usefulness of urinary 1-hydroxynaphthalene in low PAH exposure from urban air pollution and associated diseases is yet unknown.^32^ A study examined the relationship between serum biomarkers of cardiovascular disease and urinary 1-hydroxynaphthalene levels but did not investigate any significant link between serum biomarkers of inflammation and urinary 1-hydroxynaphthalene levels.^33^ Furthermore, other research investigated the relationship between urinary PAH metabolites and metabolic syndrome in non-diabetic individuals and found no significant relationship between urinary1-hydroxynaphthalene levels and metabolic syndrome and its elements.^34^ However, several investigations have found a link between 1-hydroxynaphthalene and inflammation and oxidative stress indicators^35^ as well as cardiometabolic disorders.^36,37^

PAHs are typically ingested through the lungs and can be removed by bronchial clearance.^38^ Impaired mucociliary clearance of contaminated particles may increase particle penetration into bronchial epithelial cells, where PAHs are oxidized.^38^ The majority of PAHs are eliminated from the body after a few hours of exposure. However, tiny quantities are known to be stored in body fat and the liver, which might lead to PAH bioaccumulation over time.^39^ PAHs were converted in the body to OH-PAHs and mostly excreted in the urine a few hours after exposure.^13^ Due to the short time to excretion of PAHs from the body, this study could not find an association between smoking and exercise modes. In a subgroup analysis, an association was found among non-smokers only.

In contrast, the Korean National Environmental Health Survey, a cross-sectional nationwide biomonitoring survey of 6478 participants ≥19 y of age, found a positive association with smokers.^40^ Our results indicated that participants who performed vigorous and moderate work activity had no association with those who did not. Moreover, individuals who performed vigorous and moderate recreational activities had positive significance, as did those who did not perform vigorous and moderate recreational activities. The implicit explanation can be respiratory absorption of airborne contaminants increases with increased ventilation and diffusion capacity in the lungs during aerobic activity.^41^ Although no direct evidence of increased PAH exposure during exercise has been found in human research, prior epidemiologic studies have shown that routine exercise in polluted outdoor regions or indoor locations, such as schools, can increase air pollution exposure.^42,43^ As a result, increased urinary PAH levels in those who engage in regular physical activity might be due to increased intake of outdoor and indoor PAHs during exercise in the way described above. To verify these findings, more cohort and experimental research is needed.

BMI was examined as a possible impact modulator between PAHs and diabetes. Effect modification is a biological phenomenon in which the effects of the same exposure vary depending on the characteristics of research participants.^44^ Assessing statistical interaction and effect modification is important for determining if a given characteristic's effects are synergistic or antagonistic with exposure, as well as who would benefit most from a particular intervention.^44,45^ In this study, heterogeneity of effects was detected as evidenced by the positive association between the highest quintiles of PAHs and diabetes among individuals of normal weight (BMI <25 kg/m^2^), overweight (BMI 25–29.9 kg/m^2^) and obese (BMI ≥30 kg/m^2^). There were no effects among overweight people (BMI 25–29.9 kg/m^2^) in the second quartile of PAHs.

Obesity and insulin resistance are two major risk factors for type 2 diabetes.^46^ While the mechanisms linking PAHs to diabetes are unknown, they have the potential to impact insulin resistance and promote obesity in a variety of ways. First, PAHs disrupt the endocrine system's function and damage the function of β cells.^34^ Second, animal experiments have demonstrated that oxidative stress affects glucose metabolism and insulin resistance, which is more severe in obese people.^47^ Third, animal studies have illustrated that PAH exposure causes weight gain by affecting adipose tissue lipolysis.^48^ Finally, higher PAH levels are linked to increased systematic inflammatory activity, leading to insulin resistance.^9^ These data show that PAH exposure is linked to obesity and insulin resistance, which could be the cause of type 2 diabetes.

Our research has multiple significant advantages. First, our study was the first to focus on the association between urinary PAHs and diabetes, the sample was a multi-ethnic sample of the USA, the NHANES laboratory and data collecting procedures are of excellent quality and we included the capacity to account for confounders from a general community without occupational PAH exposure. Second, we estimated individual PAH exposure using urinary PAH metabolites, indicating PAH exposure from various sources. Third, this study examined the relationship between urinary PAHs and diabetes in the general population of the USA, taking into account possible confounders like age, gender, marital status, ethnicity, education, work type, housing type, number of people in the household, vigorous work activity, moderate work activity, vigorous recreational activities, moderate recreational activities, smoking, drinking and BMI.

However, the study has several limitations. First, due to the cross-sectional study design of the NHANES, the causal inferences regarding the association between PAHs and the risk of DM cannot be proven. Second, the NHANES does not collect data on dietary exposure to PAHs, thus the proportion of dietary exposure to PAHs and the proportion of dietary PAHs in total exposure cannot be determined in those who eat grilled or charred meat regularly, especially high-fat meats.^49^ Although eating habits were assumed to be reasonably homogeneous for individuals in the same community, we did not investigate the confounding effects of dietary patterns. Third, because the NHANES does not gather data on the type of diabetes, we cannot discriminate between type 1 and type 2 diabetes. However, based on the demographic distribution of the two DM phenotypes, we estimate that the bulk of diabetic individuals in our sample have type 2 diabetes. It is also conceivable that DM may potentially produce greater PAH concentrations in the body due to impaired renal functions, reverse causation that we address but that cannot be ruled out in prevalence data.

## Conclusions

In conclusion, this population-based cross-sectional study found a positive association between urinary PAHs and DM prevalence in the US population.

## Data Availability

Data are publicly available at https://www.cdc.gov/nchs/nhanes/index.htm.
